# Clinical Options in Relapsed or Refractory Hodgkin Lymphoma: An Updated Review

**DOI:** 10.1155/2015/968212

**Published:** 2015-12-16

**Authors:** Roberta Fedele, Massimo Martino, Anna Grazia Recchia, Giuseppe Irrera, Massimo Gentile, Fortunato Morabito

**Affiliations:** ^1^Hematology and Stem Cell Transplant Unit, Azienda Ospedaliera BMM, 89100 Reggio Calabria, Italy; ^2^Biotechnology Research Unit, Azienda Sanitaria Provinciale di Cosenza, 87051 Aprigliano, Italy; ^3^Hematology Unit, Azienda Ospedaliera di Cosenza, 87100 Cosenza, Italy

## Abstract

Hodgkin lymphoma (HL) is a potentially curable lymphoma, and modern therapy is expected to successfully cure more than 80% of the patients. Second-line salvage high-dose chemotherapy and autologous stem cell transplantation (auto-SCT) have an established role in the management of refractory and relapsed HL, leading to long-lasting responses in approximately 50% of relapsed patients and a minority of refractory patients. Patients progressing after intensive treatments, such as auto-SCT, have a very poor outcome. Allogeneic SCT represents the only strategy with a curative potential for these patients; however, its role is controversial. Based on recent knowledge of HL pathology, biology, and immunology, antibody-drug conjugates targeting CD30, small molecule inhibitors of cell signaling, and antibodies that inhibit immune checkpoints are currently explored. This review will discuss the clinical results regarding auto-SCT and allo-SCT as well as the current role of emerging new treatment strategies.

## 1. Introduction

Hodgkin lymphoma (HL) is a potentially curable lymphoma with distinct histology, biological behavior, and clinical characteristics. Thomas Hodgkin first described the disorder in 1832. In the 20th century, with the realization that the disease consisted of a lymphoid malignancy, it was renamed HL. It is a relatively rare disease and accounts for approximately 10% of all malignant lymphomas, with about 9,200 estimated new cases and 1,200 estimated deaths per year in the United States [1]. The treatment of HL has evolved over the past three decades, and modern therapy is expected to successfully cure over 80% of patients [2]. Second-line salvage high-dose chemotherapy (HDC) and autologous stem cell transplantation (auto-SCT) have become the standard care for refractory/relapsed HL, leading to long-lasting responses in approximately 50% of relapsed patients and in a minority of refractory patients [3]. Disease recurrence or progression after auto-SCT is associated with very poor prognosis [4] and patients have an estimated average survival of less than 3 years [5]. However, because HL is a rare cancer that is highly curable, the development of new drugs for the treatment of HL has been very slow [6]. With growing knowledge of HL pathology, biology, and immunology, several therapeutic targets have been identified and are currently under preclinical and clinical investigation [7]. The aim of drug development in HL is not only to cure patients, but also to go further and decrease the toxic effects of therapy.

In this review, we summarize the most recent updates on the management of patients with relapsed or refractory HL and the role of novel therapeutic approaches. We also discuss the role of consolidation strategies such as HDC and auto-SCT and reduced-intensity (RIC) allogeneic stem cell transplantation (allo-SCT).

## 2. Autologous Stem Cell Transplantation

According to retrospective and prospective as well as randomized studies, HDC followed by auto-SCT can rescue 30% to 80% of relapsed/refractory HL patients [8–14].

In the BNLI trial [12], relapsed patients were treated with conventional dose mini-BEAM (carmustine, etoposide, cytarabine, and melphalan) or high-dose BEAM with auto-SCT. Both event-free survival (EFS) and progression-free survival (PFS) showed significant differences in favor of BEAM plus transplant (*p* = 0.025 and *p* = 0.005, resp.). In the GHSG trial [13], patients who relapsed after chemotherapy were randomly given four courses of mini-BEAM+dexamethasone (dexa-mini-BEAM) or two courses of dexa-mini-BEAM followed by BEAM and auto-SCT. Freedom from treatment failure (FFTF) in 3 years was significantly better for patients given BEAM and auto-SCT (55%) than for those on dexa-mini-BEAM (34%; *p* = 0.019). Overall survival (OS) of patients given either treatment did not differ significantly. Recently, the GHSG group [14] evaluated the impact of sequential HDC before myeloablative therapy. Patients with histologically confirmed, relapsed HL were treated with two cycles of dexamethasone, cytarabine, and cisplatin, and those without disease progression were then randomly divided between standard and experimental treatment arms. In the standard arm, patients received myeloablative therapy with BEAM followed by auto-SCT. In the experimental arm, patients received sequential cyclophosphamide, methotrexate, and etoposide in high doses before BEAM. Mortality was similar in both arms (20% and 18%). With a median observation time of 42 months, there was no significant difference in terms of FFTF (*p* = 0.56) and OS (*p* = 0.82) between arms. FFTF in 3 years was 62% and OS was 80%. Results demonstrated that sequential HDC did not improve outcome and was associated with more adverse events and toxicity. Based on the data presented, the authors concluded that two cycles of intensified conventional chemotherapy (DHAP) followed by HDC (BEAM) and auto-SCT are an effective and safe treatment strategy for patients with relapsed HL.

On the basis of this study, BEAM is considered the gold standard conditioning regimen for auto-SCT. However, due to drug constraints of carmustine, this drug is often replaced by a variety of agents, including fotemustine [15], bendamustine [16], and thiotepa [17].

Sweetenham et al. [18] published a retrospective analysis of 175 patients with HL who did not undergo remission after induction therapy and results were reported to the European Group for Bone Marrow Transplantation (EBMT). The 5-year actuarial OS and PFS rates were 36% and 32%, respectively, and results were very similar to those reported from single-institution series and from the Autologous Blood and Marrow Transplant Registry (ABMTR) [19]. The ABMTR series includes 122 patients with HL who have never achieved remission. The definition of failure to achieve remission differs from that in the EBMT series, in that it includes only those patients who had a documented disease progression or tissue confirmation of persistent disease in residual radiographic abnormalities. With a median follow-up of 28 months from the date of auto-SCT, the 3-year actuarial PFS and OS rates in this series were 38% and 50%, respectively. The GELTAMO Cooperative Group [20] presented the results of 62 patients treated with an auto-SCT for refractory HL. One-year transplant-related mortality (TRM) was 14%. The response rate in 3 months after auto-SCT was 52%. Actuarial 5-year time to treatment failure (TTF) and OS were 15% and 26%, respectively. The presence of B symptoms at auto-SCT was the only adverse prognostic factor significantly influencing TTF. The presence of B symptoms at diagnosis, MOPP-like regimens as first-line therapy, bulky disease at auto-SCT, and two or more lines of therapy before auto-SCT adversely influenced OS.

Tandem auto-SCT for HL has been evaluated in a small number of studies [21–24] and in the most recent guidelines from the American Society for Blood and Marrow Transplantation it is not recommended, although further studies may be warranted in high-risk patients [25].

## 3. Allogeneic Stem Cell Transplantation

Although there is relatively limited accessible data regarding the best approach for patients who relapse after an auto-SCT, the available information supports the benefit of allo-SCT versus standard therapy [25–28]. Evidence of a graft versus HL (GVHL) effect comes from the demonstration that the development of graft versus host disease (GVHD) after allo-SCT is associated with a lower relapse rate [29, 30]. Moreover, the most direct evidence for a graft versus malignancy effect comes from the disease responses to donor lymphocyte infusions (DLIs). Peggs et al. [31] assessed the impact of DLI on relapse incidence when administered for mixed chimerism and the utility of DLI as salvage therapy when given for relapse in 76 consecutive patients with multiple relapsed or refractory HL, who underwent allo-SCT that incorporated in vivo T-cell depletion. The results demonstrated the potential for allogeneic immunotherapy with DLIs both to reduce relapse risk and to induce durable antitumor responses.

Despite early data showing promisingly low relapse rates after allo-SCT, the transplantation community was not very enthusiastic about considering allo-SCT for HL patients, because of the exceedingly high nonrelapse mortality (NRM). Registry data [32, 33] has shown that allo-SCT after myeloablative conditioning results in lower relapse rates but significantly higher toxicity than auto-SCT. Although the poor results after myeloablative conditioning can be explained by the very poor risk features of heavily pretreated patients included in these early trials, high TRM has been associated with high incidence of GVHD and infections after transplantation. Results of allo-SCT can be reasonably optimized with a better patient selection and the use of targeted and less toxic therapies to achieve an adequate response for patients.

In the last years, the use of RIC has reduced NRM and improved OS [34] and the percentage of patients with refractory and relapsed HL treated using this approach has been growing steadily in Europe [35]. Robinson et al. [36] conducted a retrospective analysis of 285 patients with HL who underwent a RIC allo-SCT in order to identify prognostic factors of outcome. Eighty percent of patients had undergone a prior auto-SCT and 25% had refractory disease at transplant. NRM was associated with chemorefractory disease, poor performance status, age > 45, and transplantation before 2002. For patients with no risk factors, the 3-year NRM rate was 12.5% compared to 46.2% for patients with two or more risk factors. The use of an unrelated donor had no adverse effects on the NRM. The development of chronic GVHD was associated with a lower relapse rate. The disease progression rate in 1 and 5 years was 41% and 58.7%, respectively, and was associated with chemorefractory disease and extent of prior therapy. PFS and OS were both associated with performance status and disease status at transplant. Patients with neither risk factor had a 3-year PFS and OS of 42% and 56%, respectively, compared to 8% and 25% for patients with one or more risk factors. Relapse within 6 months of a prior auto-SCT was associated with a higher relapse rate and a lower PFS.

In the analysis by Robinson et al., the authors also identified important clinical parameters predicting transplant outcomes. RIC allo-SCT may be an effective salvage strategy for the minority of patients with good risk features who relapse after an auto-SCT, with similar outcomes for both sibling and matched unrelated donor (MUD) transplants. On the other hand, for patients with chemorefractory disease or a poor performance status, the overall outcome is poor and it is difficult to recommend RIC allo-SCT for these patients.

Burroughs et al. [37] evaluated the outcome of RIC allo-SCT for patients with relapsed or refractory HL based on different donor cell sources. Ninety patients with HL were treated with nonmyeloablative conditioning followed by allo-SCT from HLA-matched related, unrelated, or HLA-haploidentical related donors. The nonmyeloablative preparative regimen consisted in either 2-Gy total body irradiation (TBI) or combination with fludarabine 30 mg/m2/day followed by postgrafting immunosuppression with mycophenolate mofetil or cyclosporine/tacrolimus. Patients were heavily pretreated with a median of five regimens and most patients had failed auto-SCT and local radiation therapy. With a median follow-up of 25 months, the 2-year OS, the PFS, and incidence of relapsed/progressive disease were 53%, 23%, and 56% (HLA-matched related); 58%, 29%, and 63% (unrelated); and 58%, 51%, and 40% (HLA-haploidentical related), respectively. NRM was significantly lower for HLA-haploidentical related (*p* = 0.02) recipients compared to HLA-matched related recipients. There were promising results with significantly decreased risks of relapse for HLA-haploidentical related recipients compared to HLA-matched related (*p* = 0.01) and unrelated (*p* = 0.03) recipients. The incidence of acute GVHD grade III/IV and extensive chronic GVHD was 16%/50% (HLA-matched related), 8%/63% (unrelated), and 11%/35% (HLA-haploidentical related), respectively.

Raiola et al. [38] confirmed in 26 advanced HL patients the results published by the Baltimore/Seattle group [39], using haplo-mismatched marrow grafts and posttransplantation cyclophosphamide. The procedure was feasible, with a low rate of GVHD and NRM, and was associated with a durable remission in a high proportion of patients. The 4-year OS and EFS were 77% and 63%, respectively. EFS was statistically different when patients were stratified according to disease phase: 1-year PFS was 100%, 67%, and 37% for patients in complete remission (CR) (*n* = 9), partial remission (PR) (*n* = 9), or resistant disease (*n* = 8), respectively (*p* = 0.02). Actuarial survival was not statistically different in the three groups (*p* = 0.1). The cumulative incidence of NRM was 4%. The 100-day cumulative incidence of grade I and grade II–IV acute GVHD was 4% and 24%, respectively; the cumulative 3-year incidence of moderate chronic GVHD was 9%.

The Lymphoma Working Party (LWP) of the EBMT, together with the GEL/TAMO [40], undertook the largest multicenter phase II prospective clinical trial presented up to now with the objective of analyzing the NRM and other major outcome parameters after allo-SCT in relapsed/refractory HL. In this study, 92 patients with an HLA-identical sibling, a MUD, or a one antigen mismatched, unrelated donor were treated with salvage chemotherapy followed by RIC allo-SCT. Fludarabine (150 mg/m2 intravenously) and melphalan (140 mg/m2 intravenously) were used as the conditioning regimen. The addition of antithymocyte globulin was used as GVHD prophylaxis for recipients of grafts from unrelated donors. The NRM rate was 8% in 100 days and 15% in 1 year. Relapse was the major cause of failure. The PFS rate was 48% in 1 year and 24% in 4 years. The OS rate was 71% in 1 year and 43% in 4 years. The results of this study emphasize the role of RIC allo-SCT in patients with relapsed/refractory HL after auto-SCT. The plateau phase in the survival curve of the subset of patients allografted in CR indicates the existence of a clinically beneficial GVHL effect. Chronic GVHD was associated with a significantly lower relapse incidence after transplantation and consequently a significant improvement of PFS.

Recently, the LWP of the EBMT has reported [41] the results on the outcome of the second allo-SCT (allo-SCT-2) performed in one hundred and forty patients with lymphoma, of which 31% were affected by HL. Three-year PFS, OS, relapse incidence, and NRM were 19%, 29%, 58%, and 23%, respectively. PFS and OS were significantly affected by refractory disease at allo-SCT-2 and by a short interval between allo-SCT-1 and allo-SCT-2. Long-term PFS was observed in particular in patients with HL, T-cell lymphoma, and indolent lymphoma where a GVHL effect was assumed [42]. In fact, considering that, in many patients, chronic GVHD was absent after allo-SCT-1 but not after allo-SCT-2, it is possible to conclude that the second allotransplant might induce an effective allo-response in patients in which GVHD failed to appear after the first transplant. Allo-SCT-2 can result in long-term disease control in patients with lymphoma recurrence after allo-SCT-1, in particular if relapse occurs late and is chemosensitive.

## 4. Brentuximab Vedotin

The expression of CD30 by Reed-Sternberg cells (RSc) coupled with its highly restricted expression makes it an obvious target for monoclonal antibody therapy [43, 44]. Results from two clinical studies using first-generation naked anti-CD30 monoclonal antibodies in patients with relapsed HL have been disappointing, perhaps reflecting their poor antigen binding and/or effector cell activation properties [45, 46]. In an alternate strategy, the anti-CD30 antibody cAC10 was conjugated to a synthetic antimicrotubule agent, monomethyl auristatin E (MMAE), resulting in the novel immunotoxin conjugate brentuximab vedotin [47]. In a phase I dose escalation trial that enrolled 45 patients with relapsed or refractory CD30+ hematologic malignancies [48], objective responses, including 11 CRs, were observed in 17 patients and tumor regression was observed in 86% of evaluable patients. Seventy-three percent of patients in that trial had undergone auto-SCT. Brentuximab vedotin (1.8 mg/kg intravenously every 3 weeks) was subsequently evaluated in a pivotal phase 2 study of 102 patients with relapsed/refractory CD30+ HL after auto-SCT [49]. Objective responses were documented in 75% of patients, with CRs observed in 34% of patients, as determined by an independent radiology review facility. The estimated 12-month survival rate was 89% and the median PFS was 5.6 months. Adverse events associated with brentuximab vedotin were typically of grade I/II and were treated through standard supportive care. Cumulative peripheral neuropathy, the most meaningful clinical adverse effect, improved or resolved completely in 80% of patients during the study.

Median OS and PFS were estimated in 40.5 months and 9.3 months, respectively. Improved outcomes were observed in patients who achieved a CR on brentuximab vedotin, with estimated 3-year OS and PFS rates of 73% and 58%, respectively, in this group of patients [50]. Of the 34 patients who obtained CR, 16 (47%) remain progression-free after a median of 53.3 months (range, 29.0 to 56.2 months); 12 patients remain progression-free without a consolidative allo-SCT. Younger age, good performance status, and lower disease burden at baseline were characteristic of patients who achieved a CR and were favorable prognostic factors for OS.

On the basis of these studies, brentuximab vedotin has been approved for the treatment of adult patients with relapsed or refractory CD30+ HL following auto-SCT or following at least two prior therapies with auto-SCT or multiagent chemotherapy.

The randomized, double-blind, placebo-controlled, phase 3 AETHERA study [51] demonstrated that brentuximab vedotin improves PFS when given as early consolidation after auto-SCT in patients with HL with risk factors for relapse or progression after transplantation. The high risk of progression after auto-SCT is defined by the presence of primary refractory HL (failure to achieve CR), relapsed HL with an initial remission duration of less than 12 months, or extranodal involvement at the start of pretransplantation salvage chemotherapy. Compared with historical survival data for high-risk patients with HL undergoing auto-SCT, the 3-year OS rate exceeding 80% in this study is remarkable.

A recent SIE, SIES, GITMO position paper declares that there is now evidence for recommending brentuximab vedotin also in HL patients refractory to salvage chemotherapy who are auto-SCT candidates and as a consolidation strategy after auto-SCT. The use of brentuximab vedotin in HL after relapse from allo-SCT or as first-line therapy is at present only experimental [52]. The Expert Panel recommends that, in the approved indications of brentuximab vedotin treatment for HL, treatment evaluation must be performed after 4 courses, and the subsequent treatment should be determined according to the response. In patients with HL attaining a CR, either an early consolidation program including allo-SCT or brentuximab vedotin therapy continued up to 16 cycles is the approved indications. It is necessary to perform clinical trials to clarify which one of the two strategies is more appropriate. Early allo-SCT should strongly be considered in patients with HL attaining a PR. Patients not eligible for transplant should be treated with brentuximab vedotin up to a maximum of 16 cycles. In patients with HL and a stable disease, the decision to continue brentuximab vedotin should rely on a patient-centered balance between clinical benefits and risks. In patients with HL and a progressive disease, brentuximab vedotin therapy should be discontinued and patients must be enrolled in clinical trials.

## 5. Bendamustine

Bendamustine is a bifunctional alkylating agent with only partial cross-resistance to other alkylating drugs, making it an attractive agent for use in the relapsed setting [53]. Although it was developed in the 1960s and used in Germany for both HL and non-HL, it has been approved for treatment of chronic lymphatic leukemia and indolent B-cell non-HL [54] and limited data exist regarding its activity in HL patients. Moskowitz et al. [55] performed a phase II study evaluating the efficacy and toxicity of bendamustine in relapsed and refractory HL. Thirty-six patients were enrolled, and 25 patients were potentially eligible for allo-SCT. Bendamustine 120 mg/m2 was administered on days 1 and 2 of each 28-day cycle for a total of six cycles of treatment. The dose of bendamustine was reduced to 100 mg/m2 for treatment delays >5 days because of neutropenia or thrombocytopenia. The dose was further reduced to 70 mg/m2 for subsequent delays of >5 days for neutropenia or thrombocytopenia. The most common nonhematologic toxicities were fatigue (primarily grade I) and nausea (primarily grade I). Thrombocytopenia was the most common hematologic toxicity, with 20% of patients experiencing grade III or IV thrombocytopenia. The overall response rate (ORR) for the 36 patients was 53%, demonstrating that bendamustine is a good option for heavily treated patients with relapsed and refractory HL who could proceed to consolidative SCT. Zinzani et al. [56] reported two cases of patients relapsed/refractory after brentuximab vedotin were successfully treated with bendamustine indicating that patients with HL relapsed/refractory to brentuximab vedotin therapy may be chemosensitive and may obtain a good response to subsequent bendamustine treatment. Zinzani et al. [57], after these case reports, performed a retrospective study on 27 heavily pretreated patients with relapsed or refractory HL, who had all received brentuximab vedotin as their last treatment and who showed disease progression, refractory disease, or early relapse, when retreated with bendamustine. The ORR was 55.5%, with 10 of 27 patients (37.0%) obtaining a CR. In comparison, the ORR previously observed with brentuximab vedotin in the same subset of patients was much lower (18.5%).

Considering the promising results of brentuximab vedotin and bendamustine as single drugs on patients with relapsed/refractory HL and their independent mechanisms of action with manageable safety profiles, a phase I-II study was performed evaluating the safety and efficacy of brentuximab vedotin in combination with bendamustine for the treatment of patients with HL first relapse [58]. Brentuximab vedotin 1.8 mg/kg on day 1 in combination with bendamustine 90 mg/m2 on days 1 and 2 of 3-week-cycles for up to 6 cycles had a manageable safety profile with premedication. The CR rate of the combination was 82% and ORR 94%. The majority of CRs (24/28 patients) were documented after 2 cycles of combination therapy and, in these patients, stem cell mobilization and collection were performed with success. These data indicate a promising approach for maximizing responses prior SCT in relapsed/refractory HL patients after frontline therapy. Promising data were reported by O'Connor and colleagues [59] on the combination of brentuximab vedotin and bendamustine in relapsed/refractory HL and anaplastic large T-cell lymphoma.

## 6. Panobinostat and Mocetinostat

Agents that target acetylases may regulate several oncogenic pathways including cell cycle progression, cell survival, angiogenesis, and antitumor immunity. Panobinostat and mocetinostat target histone deacetylase (HDAC) and these agents may be effective in patients with HL by modulating serum cytokine levels and the expression of PD-1 on intratumoral T-cells.

Based on promising results from a phase I study that included 13 patients with relapsed HL [60], a large pivotal international phase II study was initiated. Oral panobinostat was administered at a dose of 40 mg three times per week, every week, in 21-day cycles. Dose delays and modifications for management of adverse events were permitted, but the lowest dose allowed on study was 20 mg. Efficacy was evaluated every 2 cycles by imaging studies. Surprisingly, patients were enrolled in less than one year. The median age was 32 years (range, 18–75), and the median number of prior chemotherapeutic regimens was 4 (range, 1–7). Importantly, the median time to relapse after the first auto-SCT was only 8 months, which represents a poor prognostic indicator. Moreover, 37% of the patients did not respond to their last prior therapy. Twelve patients also received prior allo-SCT.

In a phase II study, 129 patients with relapsed and refractory HL received 40 mg of panobinostat orally three times per week [61]. Treatment with panobinostat was effective as tumor reductions were seen in 74% of patients, and ORs were achieved by 35 patients (27%). Thirty patients (23%) had partial responses to treatment and fıve patients (4%) had CRs. The median duration of response was 6.9 months, and the median PFS was 6.1 months. The treatment was reasonably well tolerated with common drug-related grade I/II adverse effects as diarrhea, nausea, fatigue, vomiting, and anorexia. Common drug-related grade III/IV adverse events were thrombocytopenia, anemia, and neutropenia. The thrombocytopenia was manageable and reversible with dose hold and modification.

Considering the synergistic activity of HDAC inhibitors with other therapies [62, 63], association studies of HDAC inhibitors combined with chemotherapy, monoclonal antibodies, and small molecule inhibitors will be evaluated. A phase I study of panobinostat combined with lenalidomide in relapsed HL is ongoing [64].

The safety and efficacy of mocetinostat were recently evaluated in a phase II study in 51 patients with relapsed classical HL [65]. Mocetinostat was given orally 3 times per week (85 mg to 110 mg starting doses) for 1 year in the absence of disease progression or prohibitive toxicity. Initially, 23 patients were enrolled in the 110 mg cohort. Subsequently, because toxicity-related dose reductions were necessary in the 110 mg cohort, 28 additional patients were treated with a dose of 85 mg. The disease control rate was 35% (eight of 23 patients) in the 110 mg group and 25% (seven of 28) in the 85 mg group. Three of the 10 (30%) patients in the 85 mg group achieved partial remissions. Furthermore, grade III and IV toxicity (mainly fatigue, with no significant hematologic toxicity) was reduced to 20%. Overall, 80% of the 30 evaluable patients had some decrease in their tumor sizes. These data demonstrate that mocetinostat has a promising single-agent clinical activity with manageable toxicity in patients with relapsed classical HL.

## 7. Everolimus

The phosphatidylinositol 3-kinase/mammalian target of rapamycin (PI3K/mTOR) signaling pathway is one of the most aberrantly activated survival pathways in cancer, making it an important target for drug development [66]. Everolimus is an oral antineoplastic agent that targets this pathway, specifıcally the mTORcomplex1 (mTORC1) that has been shown to be activated in patients with HL. Everolimus not only may target the signaling pathways within the RSc but may also suppress signaling within the immune infıltrate and production of cytokines present in the tumor microenvironment [67]. Nineteen evaluable patients with relapsed HL were treated with daily doses of 10 mg everolimus, the ORR rate was 47%, and 8 patients achieved PR and 1 CR [68]. The median time to disease progression was 7.2 months. The majority of patients had received multiple previous lines of therapy and 84% of the patients had undergone a previous auto-SCT. Grade III adverse events included thrombocytopenia and anemia. Considering that several signal transduction pathways are critical for the proliferation and survival of neoplastic Hodgkin RSc, including NF-*κ*B, JAK-STAT, PI3K-AkT, and ERK [69], a combination of therapeutic approaches capable of targeting RSc along with reactive cells of the microenvironment might prolong the response duration of mTOR inhibitors to overcome chemorefractoriness.

## 8. JAK Inhibitors

The Janus kinase (JAK) and signal transducer and activator of transcription (STAT) pathway is an active mediator of cytokine signaling in the pathogenesis of solid and hematologic malignancies. The seven-member STAT family is composed of latent cytoplasmic transcription factors that are activated by phosphorylation intertwined in a network with activation that ultimately leads to cell proliferation. Aberrant activation of the JAK-STAT pathway has been demonstrated in patients with large granular lymphocytic leukemia, aplastic anemia, myelodysplastic syndrome, myeloproliferative disorders, and HL [70]. Pacritinib is an inhibitor of JAK2 kinase with preclinical activity in a variety of hematological malignancies [71]. This JAK inhibitor has been used in a phase I clinical trial in patients with relapsed or refractory Hodgkin or non-Hodgkin lymphoma of any type except Burkitt or central nervous system lymphoma [72]. Doses of 100 to 600 mg/day were tested, and treatment was well tolerated, with mostly grade I/II toxicities. Among the 34 patients' study, the ORR was 14%, including three partial remissions. In the group of patients with HL, however, none of the 14 patients had a partial remission or better. However, at least fıve of the patients with HL did benefıt from the treatment, with a decrease in the sites of active disease.

## 9. Rituximab

Rituximab has shown activity in nodular lymphocyte predominant HL. It is active in relapsed/refractory classical HL regardless of subtype or degree of CD20 expression on RS cells. Rationale of using rituximab in classic HL includes elimination of CD20+ reactive B-cells supporting RS cells, hence depriving malignant cells of survival signals and potentially increasing host immune responses [73]. In a pilot study [74], 22 patients with recurrent, classic HL who had received a minimum of two prior treatment regimens, regardless of whether H/RS cells expressed CD20, were treated with 6 weekly doses of 375 mg/m2 rituximab to selectively deplete infiltrating benign B-cells. Five patients (22%) achieved partial or complete remission that lasted for a median of 7.8 months (range, 3.3–14.9 months). Remissions were observed in patients only at lymph node and splenic sites, but not at extranodal sites, and were irrespective of CD20 expression by H/RS cells. Furthermore, systemic (B) symptoms resolved in six of seven patients after therapy. These data need to be confirmed in clinical trial.

## 10. Lenalidomide

Lenalidomide is an immunomodulatory agent with several mechanisms of action, including direct induction of apoptosis in tumor cells, antiangiogenic effects, and the modulation of immune cells, such as natural killer cells and T-cells [75]. Limited data suggest that lenalidomide has clinical activity in relapsed/refractory HL. Fehniger et al. [76] evaluated 38 relapsed HL patients with 25 mg/day of lenalidomide on days 1–21 of 28-day cycles; 33 of 38 patients had prior SCTs. The ORR to lenalidomide in the 35 evaluable patients was 17%, with one CR. Additional six patients had stable disease (SD) lasting >6 months, resulting in an overall cytostatic response rate (CR + PR + SD > 6 months) of 34%. Treatment continued until progressive disease or an unacceptable adverse event. Kuruvilla et al. [77] evaluated lenalidomide in 14 patients with relapsed or refractory HL. Two patients achieved a PR (14%), with additional seven patients having SD (50%). The median time to progression in that study was only 3.2 months, with a median OS time of 9.1 months. Böll and colleagues used lenalidomide in 42 patients [78]. Preliminary results involving the first 24 patients have been reported. Twelve patients (50%) had an objective response (11 with a PR and one with a CR), with additional eight patients achieving SD. Further studies must be made to evaluate the actual efficacy and long-lasting effect of lenalidomide.

Lenalidomide was further evaluated in HL by the GHSG in a first-line phase I combination trial for older patients [79]. The GHSG aimed to improve the ABVD regimen by replacing bleomycin with lenalidomide (AVD-Rev) to improve both efficacy and tolerability of the regimen. Patients received four to eight cycles of AVD-Rev (standard-dose AVD on days 1 and 15 of a 28-day cycle and lenalidomide daily from days 1 to 21) followed by radiotherapy. The daily lenalidomide dose for the first patient was 5 mg; maximum dose in this dose escalation trial was 25 mg. Twenty-five patients with a median age of 67 were enrolled. Sixty-eight had advanced stage disease, and 80% had B symptoms at diagnosis. After dose-limiting toxicity evaluation of 20 patients, a prespecified stopping criterion was reached and the recommended dose for a phase II trial was 25 mg. At least one grade III/IV toxicity occurred in all 22 patients who were treated at dose levels 20 and 25 mg, and 16 of those patients had a grade IV toxicity. The 1-year estimates for PFS and OS were 69 and 91%, respectively. In summary, AVD-Rev displayed high efficacy and a manageable toxicity profile in older patients with HL and should be further evaluated in phase II/III trials. In addition, a phase II trial combining lenalidomide and panobinostat in patients with relapsed or refractory HL is currently recruiting.

## 11. Anti-PD-1 Antibodies

The concept that the immune system plays a critical role in controlling and eradicating cancer and that the immune response, driven by T-lymphocytes, is closely regulated through a complicated and delicate balance of inhibitory checkpoints and activating signals is well established [80, 81]. Programmed death-1 (PD-1) is one of the main immune checkpoint receptors that, when binding its programmed death-ligand-1 (PD-L1), determines the downregulation of the T-cell effector functions, thus contributing to the maintenance of the tolerance to tumor cells. The blockade of this pathway by anti-PD-1 and anti-PD-L1 antibodies may prevent this downregulation and allows T-cells to maintain their antitumor property and ability to mediate the tumor cell death [82–84]. The genes encoding the PD-1 ligands, PD-L1 and PD-L2, are key targets of chromosome 9p24.1 amplification, a recurrent genetic abnormality in the nodular sclerosis type of HL. The 9p24.1 amplicon also includes JAK2, and gene dose-dependent JAK-STAT activity further induces PD-1 ligand transcription [85]. The complementary mechanisms of PD-1 ligand overexpression in HL suggest that this disease may have genetically determined vulnerability to PD-1 blockade. For these reasons, in a phase I study, 23 extensively pretreated patients with relapsed or refractory HL were given every 2 weeks 3 mg/kg nivolumab, a fully human monoclonal IgG4 antibody directed against PD-1 [86]. The majority of these patients had previously received an auto-SCT, and most had received previous brentuximab vedotin. Drug-related adverse events of any grade were reported in 18 (78%) of 23 patients, and grade III drug-related adverse events were reported in five (22%) patients. Of 23 patients, four (17%) had a CR, 16 (70%) had a PR, and three (13%) had SD. In 24 weeks, the rate of PFS was 86% resulting in a very high proportion of patients achieving an overall response and clinical benefit. The study shows promising results; however, larger trials are needed before introducing nivolumab in HL treatment.

A multicenter, open-label, phase Ib clinical trial is ongoing evaluating the use of the humanized IgG4 monoclonal antibody pembrolizumab (formerly MK-3475), targeting the PD-1 receptor, in relapsed or refractory HL patients who failed brentuximab vedotin treatment, with adequate performance status and organ function [87]. Pembrolizumab 10 mg/kg was administered in 15 patients intravenously every 2 weeks until confirmed tumor progression, excessive toxicity, or completion of 2 years of therapy. The drug was well tolerated with no serious adverse events, and only one patient experienced grade III pain and grade III joint swelling. The most common drug-related adverse events were grade I/II respiratory events (20%) and thyroid disorders (20%). Three patients (20%) had a CR in 12 weeks. Five additional patients (33%) had a PR as the best overall response, for an ORR of 53%. Four patients (27%) experienced progressive disease, although all 4 experienced a decrease in their overall tumor burden. In conclusion, pembrolizumab therapy appears to be safe, tolerable, and associated with clinical benefit in patients with heavily pretreated HL.

## 12. Conclusions

Auto-SCT is the standard of care for refractory/relapsed HL, leading to long-lasting responses in approximately 50% of relapsed patients and in a minority of refractory patients. Patients progressing after intensive treatments, such as auto-SCT, have a very poor outcome.

In the recent past, particularly effective novel therapies have been identified to treat these patients (Table 1). These agents have all been tested as single drugs acting on different pathways implicated in the pathogenesis of HL (Table 2), and therefore an important future approach will be to combine them with each other and with standard chemotherapies.

Up to now, brentuximab vedotin is the only FDA approved drug for the treatment of relapsed HL. There have been attempts to combine brentuximab vedotin in a pretransplant setting, either in sequential mode, that is, brentuximab vedotin as a single agent, followed by HDC, or concurrently (i.e., with bendamustine). Either way, there is an improvement in the overall response rate and complete response rate with these treatment strategies, and this may evolve with time to include brentuximab vedotin as part of the induction in pretransplant regimens.

PD1-targeted therapies, pembrolizumab and nivolumab, are becoming very good potential drugs, and most likely both will be approved in the near future.

Although these new therapies have clearly demonstrated efficacy in HL, a molecularly targeted drug achieving long-term responses with good tolerability is still lacking. Moreover, the majority of patients are young, and in this scenario we believe that allo-SCT can play an important role in selected patients.

## Figures and Tables

**Table 1:** Novel agents evaluated in relapsed/refractory HL patients after auto-SCT.

Author	Therapeutic agent(s), study design	Pts. *N*1	Pts. *N*2	Response rate	Median duration of response
Younes et al., 2010 [48]	Brentuximab vedotin, phase I	42	33	ORR = 38% CR = 24%	9.7 months
Younes et al., 2012 [49]	Brentuximab vedotin, phase II	102	102	ORR = 75% CR = 34%	20.5 months for patients in CR
Moskowitz et al., 2013 [55]	Bendamustine, phase II	35	27	ORR = 53% CR = 33%	5 months
Zinzani et al., 2015 [57]	Bendamustine, retrospective	27	27	ORR = 55.5% CR = 37%	8 months
LaCasce et al., 2014 [58]	Bendamustine + brentuximab, phases I-II	45	—	ORR = 94% CR = 82%	NR
Younes et al., 2012 [61]	Panobinostat, phase II	129	129	ORR = 27% CR = 4%	6.9 months
Younes et al., 2011 [65]	Mocetinostat, phase II	51	43	ORR = 33%	NR
Johnston et al., 2010 [68]	Everolimus, phase II	19	16	ORR = 47% CR = 5%	7.2 months
Younes et al., 2012 [72]	Pacritinib, phase I	34	14	ORR = 14%	130 days
Younes et al., 2003 [74]	Rituximab, phase II	22	18	ORR = 22%	7.8 months
Fehniger et al., 2011 [76]	Lenalidomide, phase II	38	33	ORR = 17%	15 months
Kuruvilla et al., 2008 [77]	Lenalidomide, phase II	14	10	PR = 14% SD = 50%	Median OS was 9.1 months
Böll et al., 2010 [78]	Lenalidomide phase II	42	NR	ORR = 50%	NR
Ansell et al., 2015 [86]	Nivolumab, phase I	23	18	ORR = 87% SD = 13%	PFS in 24 weeks was 86%
Moskowitz et al. 2014 [87]	Pembrolizumab, phase I	15	15	ORR = 53% CR = 20%	NR

Pts. *N*1: total number of patients; Pts. *N*2: patients who received prior auto-SCT; ORR: overall response rate; CR: complete remission; PR: partial remission; SD: stable disease; OS: overall survival; PFS: progression-free survival; NR: not reported.

**Table 2:** Competitive environment.

Agent	Indication	Development stage	Mechanism of action
Brentuximab vedotin	HL, NHL	Approved for HL and NHL	Anti-CD30 antibody-drug conjugate

Bendamustine	NHL, MM	Approved for NHL	Bifunctional alkylating agent

Panobinostat	AML, CML, breast cancer, prostate cancer, MM, idiopathic myelofibrosis, HL, NHL	Approved for MM	HDAC inhibitor

Mocetinostat	AML, solid tumors, CLL, MDS, NHL, HL	Phase II	HDAC inhibitor

Everolimus	Solid tumors, transplant rejection, HL, NHL	Approved for solid tumors and transplant rejection	mTOR inhibitor

Pacritinib	AML, myeloproliferative disorders, HL, NHL	Phase III	JAK2-inhibitor

Rituximab	NHL, CLL, rheumatoid arthritis, HL, granulomatosis, multiple sclerosis, MM	Approved for NHL, rheumatoid arthritis, granulomatosis, CLL	Anti-CD20 antibody

Lenalidomide	MDS, MM, NHL, HL, CLL	Approved for MDS, MM	Immunomodulator

Nivolumab	Melanoma, lung cancer, renal cancer, HL	Phase I	Anti-PD1 antibody

Pembrolizumab	Melanoma, lung cancer, renal cancer, HL	Phase I	Anti-PD1 antibody

HL: Hodgkin lymphoma; NHL: non-Hodgkin lymphoma; MM: multiple myeloma; AML: acute myeloid leukemia; CML: chronic myeloid leukemia; MDS: myelodysplastic syndrome; CLL: chronic lymphatic leukemia.
